# Two Guineas, Madam

**Published:** 1887-07-02

**Authors:** 


### TWO GUINEAS, MADAM.

A COUNTRY vicar of the writer's acquaintance was once in his curate days minus a sermon on Saturday afternoon. For some unremembered reason, he found it would be difficult, if not impossible, to prepare one even of ten minutes' duration. In this dilemma, his mother, who had often been his helpful providence on previous occasions, came to the rescue and volunteered not only to write a sermon, but to fit it with a suitable text. "Temptation," was her theme, and her exposition took a concrete rather than an abstract form. She explained the peculiar temptations of different classes of persons, and among the rest indicated what she considered to be the rock on which medical men's morals are most liable to suffer shipwreck. "The temptation of the medical man," she solemnly announced, "the temptation of the medical man is unnecessarily to increase the number of his visits, and to attach too great an importance to his fees." The good lady was the mother of a large and delicate family, and probably spoke from rather an extensive experience of the fraternity of physic.

The fee, it must be admitted, plays a not inconsiderable part in the drama of the doctor's life. It is his friend in more ways than one. Some people take the limited view that the doctor looks upon his guineas as the pig-jobber regards his vulgar and plebeian gains. By no means, my dear sir or madam. The fee is an indicator and a watchdog. A clever and ambitious member of the pill-box tribe can gauge his position very accurately by the amount which his patient pays him with a smiling face. Is he a family doctor, and do his clients ask for a bill of particulars at Christmas, when the total is made up of so many five-shilling visits? Then he may consider that he is yet a long way off from being regarded as the burning and shining light of his own particular neighbourhood. But if his patients pay these modest charges with "compliments and thanks," he is justified in entertaining the pleased assurance that the public look upon him as one who has already "won his spurs." Five shillings, it must be confessed, is not a very liberal honorarium for a man who has spent the six or seven years of time and the thousand pounds of money required to secure the coveted M.D. But five shillings would do very well if only it were always paid. That is where the shoe pinches the unhappy doctor. By dint of hard work and great economy he could maintain a fair position and save a modest competence before age disabled him, if only everybody or almost everybody would pay. But when a tenth, or even an eighth, or a seventh of his earnings have to be written off as bad debts, all the cream is skimmed off the milk and the remainder is diluted with water. We are really sorry for the family doctor; he is a hardworking, and often a most honourable and deserving man; and these miserable bad debts are not unfrequently the last straws that break the camel's back. We have many readers among the laity, and we do not think it unworthy of their consideration to try to improve public opinion in this respect.

But the doctor never can be said to be "truly blest" until the "two guinea" stage is reached. A family physician some years ago broke down in health so much that his mind gave way. It was pathetic to hear him repeat for the hundredth time that "he must get up and dress; his carriage was waiting at the door; he had been sent for by the Prince of Wales." Poor man, who knows how often in his student and early graduate days he had dreamt of guinea fees and court phycisianships? No doubt many a poor fellow who is thankful for eighteenpence a visit has had visions of Jacob's-ladders reaching up to the heaven of Cavendish Square, or at least to that outer court of Paradise—Harley Street. Let us shed a brotherly tear over the burst and vanished bubbles of disappointed ambition. Perhaps the real Heaven will be better than Harley Street, and the family doctor's prospect of that is probably as good as his two guinea brother's.

The fee is a watchdog as well as a yard measure. What kind of life would the half-dozen most eminent physicians and surgeons lead if they had no prohibitory scale of fees? When men and women are dying, or think they are dying, reason sometimes becomes absolutely unreasonable, and, like drowning men, even philosophers will clutch at straws. The family doctor—all honour to him for it—attends when he is sent for, and takes what he can get when he can get it. The prince of physic—if there be a prince in such a charitable profession as physic—is not so entirely compliant. He too, however, is easily moved if the occasion be such as to justify a deviation from his rules. But, generally, his fees are such as to prohibit any but those who are at least moderately well-to-do.

It is a comfort to think that, after all, as the Irishman said, " One man is as good as another and a great deal better." If everybody were rewarded according to his strict deserts many a general practitioner, both in town and country, would receive the coveted distinction of " Sir Bolus," or " Sir Gregory." We will not be so ill-natured as to insinuate that by the same rule some of the Sir Gregories would be relegated to humbler spheres. That would be heresy most irrational and unpardonable, and therefore we will not give it currency. Nevertheless, it remains true for everybody's comfort that for most people the doctor who best knows their peculiarities is the best doctor; and it may be stated as a generally accurate proposition that, putting aside cases requiring specialists, the patient who cannot be cured by his intelligent family doctor is in a fair way of not being cured at all. He had better make his will without delay. The whole point of this little discourse for the layman may be stated in a single sentence: Choose an intelligent and honest family doctor; pay him justly, and give him your confidence; and then you are pretty sure to get directly from himself or through his intervention, the very best which modern medicine is able to do for you when you require its aid.

